# Extensive/Multidrug-Resistant Pneumococci Detected in Clinical Respiratory Tract Samples in Southern Sweden Are Closely Related to International Multidrug-Resistant Lineages

**DOI:** 10.3389/fcimb.2022.824449

**Published:** 2022-03-22

**Authors:** Linda Yamba Yamba, Fabian Uddén, Kurt Fuursted, Jonas Ahl, Hans-Christian Slotved, Kristian Riesbeck

**Affiliations:** ^1^ Clinical Microbiology, Department of Translational Medicine, Faculty of Medicine, Lund University, Malmö, Sweden; ^2^ Department of Bacteria, Parasites and Fungi, Statens Serum Institut, Copenhagen, Denmark; ^3^ Infectious Diseases, Department of Translational Medicine, Faculty of Medicine, Lund University, Malmö, Sweden

**Keywords:** antimicrobial resistance (AMR), extensive drug resistance (XDR), multidrug-resistant (MDR), global pneumococcal sequence cluster, mucosal infection, respiratory tract, streptococcus pneumoniae, serotype

## Abstract

**Background/Objective:**

The frequencies of non-susceptibility against common antibiotics among pneumococci vary greatly across the globe. When compared to other European countries antibiotic resistance against penicillin and macrolides has been uncommon in Sweden in recent years. Multidrug resistance (MDR) is, however, of high importance since relevant treatment options are scarce. The purpose of this study was to characterize the molecular epidemiology, presence of resistance genes and selected virulence genes of extensively drug-resistant (XDR) (*n*=15) and MDR (*n*=10) *Streptococcus pneumoniae* detected in clinical respiratory tract samples isolated from patients in a southern Swedish county 2016-2018. With the aim of relating them to global MDR pneumococci.

**Methods:**

Whole genome sequencing (WGS) was performed to determine molecular epidemiology, resistance genes and presence of selected virulence factors. Antimicrobial susceptibility profiles were determined using broth microdilution testing. Further analyses were performed on isolates from the study and from the European nucleotide archive belonging to global pneumococcal sequence cluster (GPSC) 1 (*n=*86), GPSC9 (*n=*55) and GPSC10 (*n=*57). Bacteria were analyzed regarding selected virulence determinants (pilus islet 1, pilus islet 2 and Zinc metalloproteinase C) and resistance genes.

**Results:**

Nineteen of 25 isolates were related to dominant global MDR lineages. Seventeen belonged to GPSC1, GPSC9 or GPSC10 with MDR non-PCV serotypes in GPSC9 (serotype 15A and 15C) as well as GPSC10 (serotype 7B, 15B and serogroup 24). Pilus islet-1 and pilus islet-2 were present in most sequence types belonging to GPSC1 and in two isolates within GPSC9 but were not detected in isolates belonging to GPSC10. Zinc metalloproteinase C was well conserved within all analyzed isolates belonging to GPSC9 but were not found in isolates from GPSC1 or GPSC10.

**Conclusions:**

Although MDR *S. pneumoniae* is relatively uncommon in Sweden compared to other countries, virulent non-PCV serotypes that are MDR may become an increasing problem, particularly from clusters GPSC9 and GPSC10. Since the incidence of certain serotypes (3, 15A, and 19A) found among our MDR Swedish study isolates are persistent or increasing in invasive pneumococcal disease further surveillance is warranted.

## Introduction

*Streptococcus pneumoniae* is a commensal of the human respiratory tract and its primary niche is the nasopharynx. Dissemination from the nasopharynx can lead to different types of pneumococcal disease including pneumonia, acute otitis media and sinusitis, of which the bacterial species is considered the most common etiology (Weiser et al., 2018). *S. pneumoniae* also causes invasive pneumococcal disease (IPD), characterized as meningitis, sepsis, and bacteriemic pneumonia that contributes to a high morbidity and mortality globally (Troeger et al., 2018). The most important virulence factor of the pneumococcus is the capsule and around 100 different serotypes have been identified (Ganaie et al., 2020). The first conjugated pneumococcal polysaccharide vaccine PCV7 was introduced in the year of 2000 in the US (covering serotypes 6B, 7F, 9V, 14, 18C, 19F and 23F). Serotype replacement then led to the development of higher valency vaccines, and PCV10 (PCV7 serotypes with addition of 1, 4 and 5) and PCV13 (PCV7 serotypes with addition of 1, 3, 4, 5, 6A and 19A) are mainly administrated. In southern Sweden, PCV7 was introduced in the child immunization program in 2009. PCV10 was implemented one year later with a shift to PCV13 in 2014, and a further change back to PCV10 in 2019.

In a meta-analysis performed by (Andrejko et al., 2021) on 312,783 pneumococci isolated from children, it was shown that the implementation of PCV in child immunization campaigns has resulted in an absolute significant decrease of penicillin non-susceptible pneumococci (PNSP). This reduction was mainly hypothesized to be attributed to reduction of non-susceptible vaccine serotypes (VT), suggesting the possibility to use vaccines also in control of antimicrobial resistance. Local changes with increased antimicrobial resistance due to serotype replacement is, however, a phenomenon to be aware of as has been observed in Sweden regarding IPD isolates in the post-PCV era. An increase of PNSP from 3.3% in 2007 to 5.6% in 2013-2016 has been observed, primarily due to the increase of non-vaccine types (NVT) PNSP (Naucler et al., 2017). Serotype switch events in already multidrug-resistant (MDR; nonsusceptibility to 3 or more different antimicrobial classes) VT lineages is another way for new clones to expand after vaccine implementation. Examples of serotype switch events have been noted in the post PCV era but the general survival rate of the resulting strains in the population is to be followed through further studies (Savinova et al., 2020; Scherer et al., 2021).

In a study by Gladstone et al. (2019) global pneumococcal sequence clusters (GPSC) were defined as a new tool for epidemiological analysis of *S. pneumoniae* in the post PCV era. It was shown that some GPSCs have an increased accumulation of MDR isolates as for example GPSC1. Close surveillance of the GPSCs is of importance for the evaluation of effect of PCVs on the overall antimicrobial resistance (AMR) within different lineages.

The pattern of non-susceptibility to common antibiotics among pneumococci vary greatly across the globe (Andrejko et al., 2021). In Sweden, AMR to penicillin and macrolides has been low in later years compared to other European countries. The European center of disease control (ECDC) estimated that Sweden, in 2019, had 6.5% PNSP and 6.5% macrolide non-susceptible invasive *S. pneumoniae* compared to the EU average of 12.1 and 14.5% (ECDC, 2020a; ECDC, 2020b). During the years 2016-2018 *S. pneumoniae* detected in clinical respiratory tract samples in Skåne County, southern Sweden, were investigated regarding serotype distribution and antimicrobial susceptibility. Of the 2,131 included isolates, 11% were PNSP and 8% macrolide non-susceptible. Furthermore, 7% were MDR and 2% were extensively drug resistant (XDR; nonsusceptibility to 5 or more different antimicrobial classes) (Golden et al., 2015; Uddén et al., 2021). MDR and XDR isolates included both VT and NVT pneumococci (Uddén et al., 2021).

The purpose of this study was to do whole genome sequencing (WGS) on pneumococcal MDR isolates detected in Skåne County between 2016-2018 with the aim of studying their resistance genes and molecular epidemiology in a global context. We present data on GPSC, resistance genes and selected virulence genes. We also show data regarding the virulence factor zinc metalloproteinase C (ZmpC) and the presence of PI-1 and PI-2 islets among different MDR GPSC as possible contributors to virulence or increased transmission among pneumococci.

## Materials and Methods

### Streptococcus pneumoniae Isolates

In our recent study, a total of 2,131 pneumococci were detected among clinical respiratory tract samples during 18 months in 2016-2018 (Uddén et al., 2021). Serotyping was performed as described in (Uddén et al., 2018). Briefly a multiplex polymerase chain reaction (PCR) scheme in combination with latex agglutination and the Quellung reaction was used. Of the 1,858 isolates that were available for further analyses, 26 isolates were classified as XDR based on non-susceptibility to ≥5 antimicrobial classes during screening with disk diffusion tests (oxacillin, erythromycin, clindamycin, tetracycline, trimethoprim-sulfamethoxazole and norfloxacin) and a gradient test for benzylpenicillin (Etest^®^; BioMérieux, Marcy-l’Étoile, FR), and were included in the current study. Minimum inhibitory concentrations (MIC) of the currently studied isolates were confirmed with broth microdilution (BMD) (Sensititre Streptococcus STP6F AST Plate; Thermo Fisher Scientific, Waltham, MA) and interpreted according to breakpoints provided by EUCAST 2021 to determine MDR (*n=*10) or XDR (*n*=16) phenotype (Uddén et al., 2021). One of 26 isolates was, however, excluded based on low quality of WGS, and not included in further analyses.

### Whole Genome Sequencing

Illumina sequencing described by (Kavalari et al., 2019) was used for WGS. Species identification was performed using ribosomal multi locus sequence typing (rMLST) provided on PubMLST (https://pubmlst.org) (Jolley et al., 2018). Capsular loci were analyzed to determine capsular genotype using the PneumoCaT tool (Kapatai et al., 2016).

### Molecular Epidemiology and Resistance Genes

Assembled genomes were uploaded to PathogenWatch (https://pathogen.watch) to assign MLST and GPSC (Jolley et al., 2018). Corresponding clonal complex (CC) of the detected sequence types, and additional epidemiological information regarding GPSC and MLST were acquired from the Global Pneumococcal Sequencing Project database (https://www.pneumogen.net/gps/GPSC_lineages.html, accessed 1-07-2021) and PubMLST. Clonal complexes were assigned by a single locus threshold (6/7) (Gladstone et al., 2019). PathogenWatch was additionally used to screen for antimicrobial resistance genes (*ermB, mefA, tetM*) and mutations (*folA/folP, gyrA/parC*) conferring resistance to commonly used antibiotics for pneumococcal disease and mosaic penicillin binding protein (PBP) profiles conferring resistance to β-lactam antibiotics in pneumococci (Li et al., 2017; Gladstone et al., 2019).

### Determination of Virulence Factors

Genes encoding the virulome were screened for using NCBI Genome Workbench version 3.6.0 and BlastN was performed for comparison of nucleotide sequences. Selected sequences for virulence genes of interest related to *S. pneumoniae* were collected from the Virulence finder database nucleotide dataset_B (downloaded 4/8-2021) (Weiser et al., 2018; Liu et al., 2019). Additional sequences were selected for detection of pilus islet 1 (PI-1) (GenBank accession numbers: EF560625–EF560637) and pilus islet 2 (PI-2) (GenBank accession number: EU311539.1) (Bagnoli et al., 2008; Moschioni et al., 2008). A 95% identity and 80% coverage were used for identification of a sequence (Kavalari et al., 2019).

### Phylogeny, Resistance Determinants and Selected Virulome of Isolates Within GPSC1, GPSC9 and GPSC10

Three phylogenetic trees were constructed with the sequenced isolates belonging to GPSC1, GPSC9 and GPSC10 together with additional reference isolates described by Gladstone et al. (Gladstone et al., 2019) provided from ENA (https://www.ebi.ac.uk/ena/browser/home) (ENA run accessions in **Supplemental Material**). The isolates were selected to represent different sequence types within GPSC1 (*n=*79), GPSC9 (*n=*50) and GPSC10 (*n=*52). MinTyper 1.0 were used for the SNP distance matrices and creation of the phylogenetic trees (Hallgren et al., 2021). The trees were rooted to concatenated sequences of different reference strains, which are specified in each figure. The phylogenetic trees were visualized using Interactive tree of life (iTol v.5) (Letunic and Bork, 2021). Assembled genomes of the ENA isolates were downloaded from PathogenWatch together with metadata including PBP-profiles, resistance genes (*ermB, mefA, tetM*) and mutations (*folA/folP*) for the isolates (date 8/8-2021). The presence of selected virulence factors (ZmpC, PI-1 and PI-2) was analyzed as previously described.

## Results

### Three Different Global Pneumococcal Sequence Clusters Dominated in the Catchment Area

Included isolates were serotyped and assigned to MLST, GPSC and CC by extraction from WGS data (**Table 1**). All isolates (*n*=25) were identified as *S. pneumoniae* using rMLST. Three different GPSC dominated among the samples, GPSC1 (*n=*7), GPSC9 (*n=*5) and GPSC10 (*n=*5). The dominating serotypes were 15A (*n=*4), 19A (*n=*3), 19F (*n=*3), 35B (*n=*3) and in total 10 different serotypes (3, 6B, 7B, 11A, 15A, 15B, 15C, 19A, 19F and 35B) and 1 serogroup (24) were identified and in general consistent with WGS. Isolates belonging to the same GPSC often carried identical resistance genes and closely related PBP profiles.

**Table 1:** XDR and MDR isolates were closely related to international MDR lineages.

Isolate	Year	Age	Sample type	Serotype	GPSC^a^	MLST	CC	Resistance	PCV coverage				Virulence		
ID		Years		Phenotype/Genotype				Profile	PCV10	PCV13	PCV15	PCV20	*PI-1*	*PI-2*	*zmpC*
1	2017	4	Nasopharynx	19F/19F	**1**	236^b^	–	XDR	+	+	+	+	+	+	–
2	2017	34	Nasopharynx	3/3	**1**	271^b^	CC320	XDR	–	+	+	+	+	+	–
3	2018	2	Nasopharynx	19A/19A	**1**	320	CC320	XDR	–	+	+	+	+	+	–
4	2018	0	Conjunctiva	19A/19A	**1**	320	CC320	XDR	–	+	+	+	+	+	–
5	2016	1	Nasopharynx	19F/19F	**1**	2920^b^	–	XDR	+	+	+	+	+	+	–
6	2018	42	Nasopharynx	19A/19A	**1**	4768	CC320	XDR	–	+	+	+	+	+	–
7	2017	88	Nasopharynx	19F/19F	**1**	8359	–	XDR	+	+	+	+	+	+	–
8	2017	37	Nasopharynx	15A/15A	**9**	63^c^	CC63	MDR-4	–	–	–	–	–	–	+
9	2017	3	Nasopharynx	15A/15A	**9**	63^c^	CC63	MDR-4	–	–	–	–	–	–	+
10	2017	7	Sputum	15C/15C	**9**	782^c^	CC63	XDR	–	–	–	–	–	–	+
11	2017	89	Sputum	15A/15A	**9**	3816^c^	CC63	MDR-4	–	–	–	–	–	–	+
12	2017	32	Sputum	15A/15A	**9**	3816^c^	CC63	MDR-3	–	–	–	–	–	–	+
13	2018	1	Nasopharynx	24/24	**10**	230^d^	CC230	MDR-4	–	–	–	–	–	–	–
14	2017	2	Middle ear	15B/15B	**10**	4253^d^	CC230	MDR-4	–	–	–	+	–	–	–
15	2017	9	Sputum	24/24	**10**	6227^d^	CC230	MDR-4	–	–	–	–	–	–	–
16	2017	1	Nasopharynx	7B/7B	**10**	Novel1	–	MDR-4	–	–	–	–	–	–	–
17	2018	27	Nasopharynx	7B/7B	**10**	Novel1	–	MDR-4	–	–	–	–	–	–	–
18	2017	2	Nasopharynx	15C/15C	**16**	83^e^	CC81	XDR	–	–	–	–	–	–	–
19	2017	1	Nasopharynx	11A/11A	43	8605	–	XDR	–	–	–	+	–	–	–
20	2017	68	Sputum	6B/6E	**47**	2040^f^	CC315	MDR-3	+	+	+	+	+	–	–
21	2016	41	Conjunctiva	NT/NT	**81**	4149	–	XDR	–	–	–	–	–	–	–
22	2017	68	Sputum	35B/35B	91	373	–	XDR	–	–	–	–	–	–	–
23	2017	58	Bronchi	35B/35B	91	373	–	XDR	–	–	–	–	–	–	–
24	2017	0	Nasopharynx	35B/35B	91	373	–	XDR	–	–	–	–	–	–	–
25	2017	0	Nasopharynx	6B/6E	**115**	135	CC1348	XDR	+	+	+	+	+	–	–
									**20%**	**36%**	**36%**	**44%**	**36%**	**28%**	**20%**

The major GPSC identified were GPSC1 (n=7), GPSC9 (n=5) and GPSC10 (n=5) and 21/25 isolates belong to GPSC known to carry a high degree of resistance. Most of the isolates (13/25) were PMEN clones or single locus variants (SLV) of PMEN clones. Major clonal complexes identified were CC320 (n=4), CC230 (n=3) and CC63 (n=5). Some isolates were reclassified as MDR due to changes in resistance pattern upon BMD testing, and updated breakpoints by EUCAST in 2019, but 15/25 showed an XDR resistance profile. PCV coverage of the sequenced isolates was low; 20% for PCV10 and 36% for PCV13. Major non-PCV serotypes detected include MDR serotype 15A,15C, serogroup 24, 7B and 35B. Additional coverage would not be increased with the use of PCV15 and only slightly with the use of PCV20, 44% with addition of serotype 11A and 15B. Pilus islet 1 (PI-1) and pilus islet-2 were present among 36% and 28% of the isolates respectively. PI-1 and PI-2 was carried by all isolates belonging to GPSC1. Zinc metalloproteinase C (ZmpC) was only present among isolates belonging to GPSC9 20% (n=5/25).

a- Bold - >2.5 in mean no of resistant classes in GPSC.

b- Identical or SLV of PMEN Taiwan19F-14 ST236.

c- Identical or SLV of PMEN Sweden15A-25 ST63.

d- Identical or SLV of PMEN Denmark14-32 ST230.

e- SLV of PMEN Spain23F-1 ST81.

f- SLV of PMEN Poland6B-20 ST315.

GPSC, Global pneumococcal sequence cluster; MLST, Multi locus sequence type; CC, Clonal complex (defined as 6/7 identical alleles); XDR, Extensively drug resistant; MDR, Multidrug resistant; PMEN, Pneumococcal molecular epidemiology network.

### ZmpC and Pilus Islets Were Present Among Only a Fraction of Isolates

The isolates were analyzed for virulence factors known to be important for pneumococcal pathogenesis and colonization. Selected virulence factors, ZmpC and pilus islets, were present among only a selection of the isolates **(** **Table 1** **)**. The virulence gene *zmpC* was only carried by the 5 isolates belonging to GPSC9. PI-1 was present in 36% (9/25) of the isolates and PI-2 in 28% (7/25) of the isolates. All isolates belonging to GPSC1 carried both PI-1 and PI-2 (*n=*7). All isolates were equipped with *ply*, *lytA, lytC, cbpE, pavA, hysA, eno, piuA, psaA, cppA, htrA* and *tig/ropA* that are common virulence genes.

### High Correlation Between Genotypic and Phenotypic Antimicrobial Susceptibility Among MDR Pneumococci

All the isolates (*n=*25) were selected for further investigation including MIC test using BMD and WGS (**Table 2**). All isolates harbored *ermB* and *tetM*, and a minority (*n=*7) also carried *mefA* belonging to GPSC1. Trimethoprim-sulfamethoxazole resistance was observed in 15/25 isolates, although 24/25 carried genes related to antibiotic resistance to sulfamethoxazole with an amino acid insertion in *folP*. The genotype correspondence with phenotype regarding β-lactam resistance was high with 23/25 isolates, having a benzylpenicillin MIC corresponding within 1 dilution step to their predicted MIC in relation to their PBP profile. Two isolates harboring *ermB* were susceptible to clindamycin upon testing with BMD, and one isolate was susceptible to tetracycline although harboring *tetM*. Two isolates harbored *cat* with phenotypic resistance to chloramphenicol. None of the included isolates carried mutations within *gyrA/parC* and were all susceptible to levofloxacin (**Supplemental Material**). All included isolates were susceptible to linezolid, vancomycin and meropenem (**Supplemental Material**). Based upon MICs determined with BMD, 10/25 isolates did not exhibit XDR phenotypes, because they were reclassified as susceptible to trimethoprim-sulfamethoxazole following updated EUCAST breakpoints in 2019.

**Table 2:** Phenotypic and genotypic antimicrobial susceptibility correspondence was high.

Isolate	β-lactam antibiotics					Macrolide/Lincosamide			Tetracycline		Trimethoprim		Chloramphenicol	
ID	PBP profile	Benzylpenicillin		Cefotaxime			Ery	Cli			Sulfamethoxazole			
	1a-2b-2x	Inferred MIC	BMD MIC	Inferred MIC	BMD MIC	*ermB/mefA*	BMD MIC	BMD MIC	*tetM*	BMD MIC	*folA/folP*	BMD MIC	*cat*	BMD MIC
1	13-16-47	2	1 I	1	0.25 S	+/+	>2 R	>1 R	+	>8 R	+/+	4 R	–	2 S
2	17-16-47	2	1 I	1	1 I	+/+	>2 R	>1 R	+	>8 R	+/+	>4 R	–	4 S
3	13-11-16	4	4 R	2	2 I	+/+	>2 R	>1 R	+	>8 R	+/+	4 R	–	4 S
4	13-11-16	4	4 R	2	2 I	+/+	>2 R	>1 R	+	>8 R	+/+	>4 R	–	4 S
5	13-16-New	4	4 R	8	2 I	+/+	>2 R	>1 R	+	>8 R	+/+	>4 R	–	8 S
6	13-11-16	4	4 R	2	2 I	+/+	>2 R	>1 R	+	>8 R	+/+	4 R	–	4 S
7	13-14-20	4	4 R	1	1 I	+/+	>2 R	>1 R	+	>8 R	+/+	>4 R	–	4 S
8	New-7-138	0.5	0.5 I	0.5	0.5 S	+/-	>2 R	>1 R	+	>8 R	-/+	1 S	–	4 S
9	24-27-28	0.25	0.12 I	0.12	0.12 S	+/-	>2 R	>1 R	+	>8 R	-/-	≤0.5 S	–	4 S
10	17-53-36	2	0.5 I	1	0.25 S	+/-	>2 R	>1 R	+	>8 R	-/+	4 R	–	4 S
11	24-27-13	0.25	0.25 I	0.25	0.12 S	+/-	>2 R	>1 R	+	>8 R	-/+	1 S	–	4 S
12	24-27-13	0.25	0.12 I	0.25	0.12 S	+/-	>2 R	>1 R	+	≤1 S	-/+	1 S	–	4 S
13	New-15-22	0.5	1 I	0.12	0.12 S	+/-	>2 R	>1 R	+	>8 R	-/+	≤0.5 S	–	8 S
14	New-15-367	0.25	0.25 I	0.12	0.12 S	+/-	>2 R	>1 R	+	>8 R	-/+	1 S	–	≤1 S
15	17-15-22	0.5	0.5 I	0.12	0.12 S	+/-	>2 R	>1 R	+	>8 R	-/+	≤0.5 S	–	2 S
16	17-144-8	2	2 I	0.5	1 I	+/-	>2 R	>1 R	+	>8 R	-/+	1 S	–	4 S
17	17-144-8	2	0.25 I	0.5	1 I	+/-	>2 R	>1 R	+	>8 R	-/+	1 S	–	4 S
18	15-12-18	2	4 R	1	1 I	+/-	>2 R	>1 R	+	>8 R	+/+	>4 R	+	16 R
19	7-12-135	0.25	0.25 I	0.12	0.12 S	+/-	>2 R	>1 R	+	>8 R	+/+	4 R	–	4 S
20	New-53-35	0.5	0.25 I	0.25	0.25 S	+/-	>2 R	0.25 S	+	>8 R	-/+	1 S	–	4 S
21	25-7-56	2	2 I	1	1 I	+/-	>2 R	>1 R	+	>8 R	+/+	4 R	–	4 S
22	7-1-New	0.25	0.25 I	0.12	0.12 S	+/-	>2 R	>1 R	+	>8 R	+/+	2 I	–	2 S
23	7-1-242	0.25	0.5 I	0.12	0.12 S	+/-	>2 R	>1 R	+	>8 R	+/+	4 R	–	4 S
24	7-1-242	0.25	0.25 I	0.12	0.12 S	+/-	>2 R	>1 R	+	>8 R	+/+	4 R	–	4 S
25	8-67-103	0.25	0.25 I	0.25	0.25 S	+/-	>2 R	>1 R	+	>8 R	+/+	>4 R	+	16 R

Similar patterns of resistance were observed based on the GPSC. GPSC1 isolates (ID 1-7) showed a higher penicillin MIC (1-4 mg/ml), carriage of ermB/mefA/tetM as well as mutations conferring resistance to co-trimoxazole. GPSC9 isolates (ID 8-12) had a lower grade of penicillin nonsusceptibility (MIC 0.12-0.5 mg/ml) and carried ermB and tetM. One isolate (ID 10) was also resistant to co-trimoxazole with the folA I100L mutation and amino acid insertions in folP 57-70. Isolates belonging to GPSC10 (ID 13-17) had a low to high grade of penicillin nonsusceptibility (MIC 0.25-2 mg/ml) and carried ermB and tetM. Remaining isolates were of varied resistance patterns but were all non-susceptible to penicillin and macrolides.

### Related Resistance Genes and Presence of Selected Virulence Genes Among Closely Associated Clones Within GPSC1, GPSC9 and GPSC10

**Figure 1:**
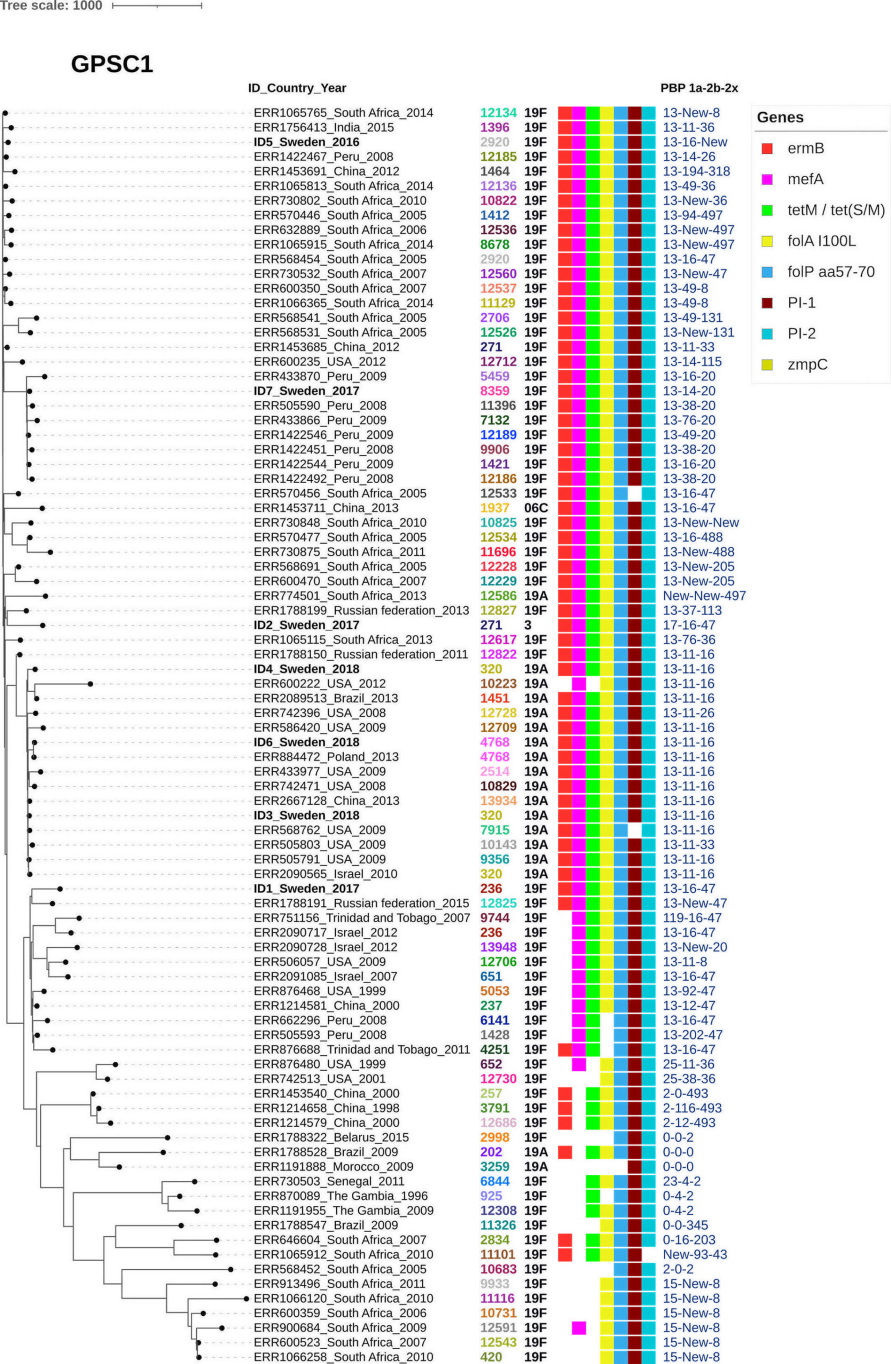
Dual presence of both Pilus islet 1 and 2 is common within GPSC1. GPSC1 tree including 86 isolates showing ID number, country and year followed by serotype, MLST, gene presence and PBP-profile (1a-2b-2x). As a reference isolate ID 1 was used. Almost all isolates (83/86) carry both Pilus islet 1 (PI-1) and Pilus islet 2 (PI-2). The three isolates that were negative for PI-1 or PI-2 however contained larger fragments of the corresponding pilus islets. All the Swedish isolates and many of the included (55/86) carry dual macrolide resistance mechanisms with both *ermB* and *mefA*. Regarding the PBP profile PBP-1a allele 13 is common as well as the profile 13-11-16. Zinc metalloproteinase C (ZmpC) was not found among either of the isolates included.

**Figure 2:**
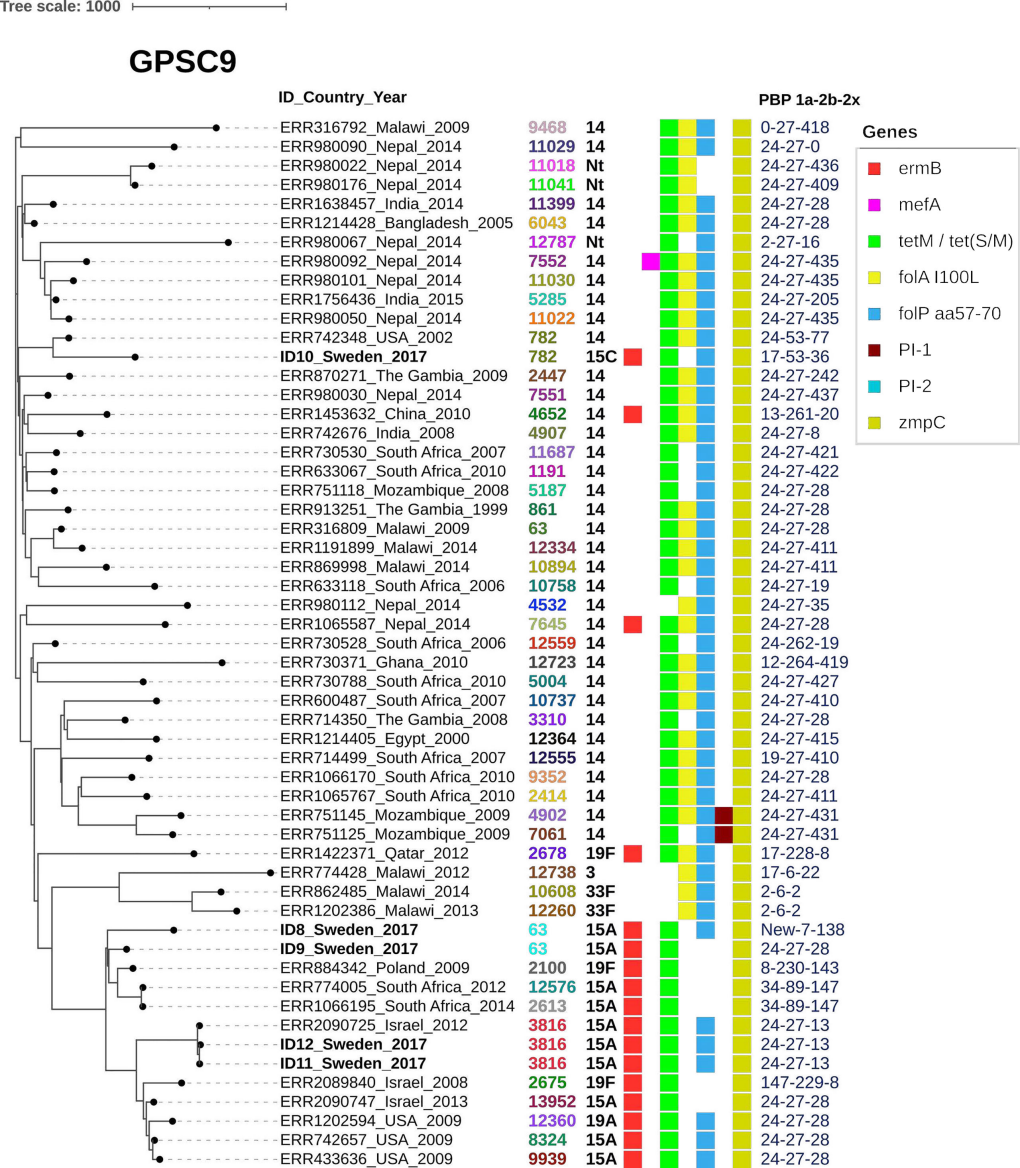
Zinc metalloproteinase C is highly preserved among isolates belonging to GPSC9. GPSC9 tree including 55 isolates showing ID number, Country and Year followed by serotype, MLST, gene presence and PBP-profile (1a-2b-2x). As a reference isolate ID 9 was used. All the Swedish isolates and all the included 100% harbor the gene for zinc metalloproteinase C (*zmpC*). Macrolide resistance gene *ermB* was mostly present in a subclade with serotype 19A, 19F and 15A isolates. Most of the Swedish isolates (4/5), belonged to this subclade with one exception being the ST782 serotype 15C isolate more closely related to other pneumococci within GPSC9. This 15C isolate diverged in both serotype and PBP profile from other closely related pneumococci. Regarding the PBP profile PBP-1a allele 24 is common within the GPSC among our included isolates. Only two isolates carried Pilus islet 1 (PI-1) that are closely related from Mozambique. Pilus islet-2 was not found among either of the isolates included.

**Figure 3:**
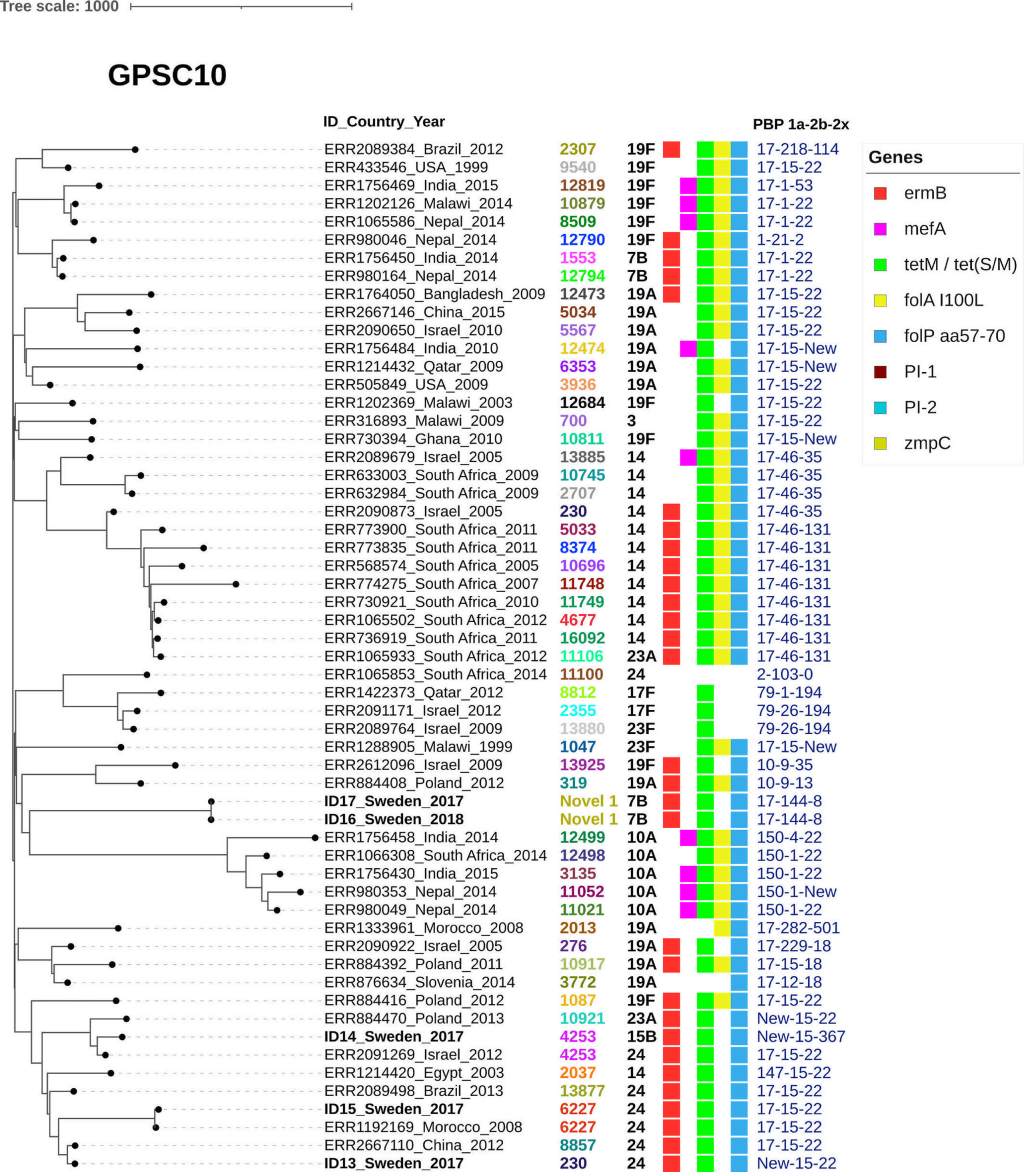
GPSC10 lack both pilus islets and ZmpC. GPSC10 tree including 57 isolates showing ID number, Country and Year followed by serotype, MLST, gene presence and PBP-profile (1a-2b-2x). As a reference isolate ID 13 was used. All the Swedish isolates and all the included 100% harbored the zink metalloproteinase C gene (*zmpC*). Macrolide resistance mechanism *ermB* was mostly present in different sub-clades with serogroup 24, serotype 14 or 7B isolates. Regarding the PBP profile PBP-1a allele 17 was common within the GPSC among our included isolates. None of the isolates carried either Pilus islet 1, Pilus islet 2 or *zmpC*.

To study selected virulence factors of interest and resistance genes, GPSC specific phylogenetic trees were constructed together with metadata. PI-1 and/or PI-2 was present among all the included isolates belonging to GPSC1 (**Figure 1**) but was only found in two isolates (PI-1) belonging to GPSC9 (**Figure 2**). No isolates belonging to GPSC10 carried PI-1 or PI-2 (**Figure 3**). All isolates belonging to GPSC9 carried *zmpC* but none of the GPSC1 or GPSC10 isolates carried the gene. Isolates that were closely related in the SNP tree carried similar PBP profiles, serotypes, and resistance genes in all the GPSC.

## Discussion

WGS was performed on clinical MDR isolates detected in Skåne County, southern Sweden during 18 months in 2016-2018. They belonged to dominant GPSCs that carry high frequencies of antibiotic resistance, namely GPSC1, GPSC9 and GPSC10 (Gladstone et al., 2019).

GPSC1 is globally dominated by serotype 19A and 19F (Gladstone et al., 2019). After the introduction of PCV7/PCV10, including serotype 19F, in child immunization programs, an increased incidence of IPD caused by MDR serotype 19A CC320, GPSC1, was observed (Schroeder et al., 2017; Cassiolato et al., 2018). In contrast, introduction of PCV13 has decreased the incidence of IPD related to serotype 19A CC320 (Isturiz et al., 2017; Schroeder et al., 2017). Serotype 19A is, however, persisting both in Skåne County and among IPD cases in Sweden, 11% in 2020 (Public Health Agency of Sweden, 2021; Uddén et al., 2021). Of note is also that a variant of serotype 19A CC320 have been associated with PCV13 failures and breakthroughs in Ireland (Corcoran et al., 2021).

Serotypes 19A and 19F were the most common serotypes in GPSC1 in our study with one exception: a serotype 3 ST271 isolate. In 2015-2017, three serotype 3 ST271 MDR isolates were detected in two different states in the US and the serotype switch within the ST271 was described by Scherer *et al.* (Scherer et al., 2021). Two additional serotype 3 ST271 pneumococci are also described in PathogenWatch, isolated in South Africa 2013. The presence of an additional similar isolate in Sweden 2017, carrying identical resistance genes and PBP profile, may indicate global spread of the clone. The finding of this isolate, that is closely related to the widely distributed 19F ST271 clone, is of concern since the use of PCV13 has not reduced serotype 3 IPD incidence in Sweden (Naucler et al., 2017; Public Health Agency of Sweden, 2021). Post PCV13 introduction in many Swedish counties, *S. pneumoniae* serotype 3 has remained the most common serotype causing IPD in Sweden, accounting for 15% of cases in 2020 (Public Health Agency of Sweden, 2021).

All currently studied GPSC1 isolates carried both PI-1 and PI-2, conferring increased adherence to host cells (Barocchi et al., 2006; Bagnoli et al., 2008). Our findings are in concordance with other studies in which the presence of PI-1 has been linked to MDR lineages with penicillin resistance, exemplified by CC320 that was also present among our MDR isolates (Dzaraly et al., 2020). In addition, GPSC1 and, specifically, CC320 have been shown to carry both PI-1 and PI-2 (Dzaraly et al., 2020; Nagaraj et al., 2021). Taken together the presence of MDR GPSC1 still warrants surveillance to follow serotype switch variants and expansion of MDR following PCV10 reintroduction in 2019 as previously discussed (Uddén et al., 2021).

GPSC10 has been described to be of concern regarding both multidrug resistance and serotype replacement in the post-PCV era (Nagaraj et al., 2021). Several isolates in our study belonged to GPSC10, including some NVTs including 7B, 15B and serogroup 24. None of these serotypes are included in currently available PCVs, and to the best of our knowledge only 15B will be included in a future multivalent PCV (Greenberg et al., 2018; Essink et al., 2020).

GPSC9 has mostly been dominated by VT 14 but serotype 15A was the most common serotype found in our study. Serotype 15A has also increased in several parts of the world following the implementation of PCV13 (van der Linden et al., 2015; Nakano et al., 2016; Wu et al., 2020), and it is not targeted by any PCV currently in development to our knowledge (Greenberg et al., 2018; Essink et al., 2020). In both Norway and the US serotype 15A has increased in the post PCV era or been related to MDR IPD cases (Beall et al., 2018; Siira et al., 2020). In the US CC63, belonging to GPSC9, was also found to be the main contributor to MDR invasive 15A isolates in 2015-2016 (Beall et al., 2018). In 2020, serotype 15A was one of the more common serotypes (7%) among IPD cases in Sweden and in 2016 serotype 15A together with 23B were the main contributors to MDR IPD (Naucler et al., 2017; Public Health Agency of Sweden, 2021). This NVT was also common among respiratory tract isolates and frequently MDR in Skåne County 2016-2018 (Uddén et al., 2021). Although the currently studied isolates are not from IPD cases, our results indicate that serotype 15A, GPSC9 MDR pneumococci are present in the population and is likely of importance regarding invasive pneumococcal disease also in Sweden.

Of the isolates detected in Skåne County, the z*mpC* gene was only present in GPSC9 (*n=*5/25). The gene was also present among all GPSC9 isolates included from ENA. The protease encoded by *zmpC* cleaves human matrix metalloproteinase 9 and has, in mouse pneumonia models, been correlated to increased virulence in comparison to knockout strains (Oggioni et al., 2003) and inhibit neutrophil influx to the lung by cleavage of P-Selectin Glycoprotein 1 (Surewaard et al., 2013). Other targets of the protease include syndecan-1 and membrane-associated mucin MUC16 that are shed from the epithelium by the protease, increasing virulence (Chen et al., 2007; Govindarajan et al., 2012). ZmpC has in an observational study also been associated to a more severe clinical presentation among IPD patients with increased rates of ICU admission (Cremers et al., 2014). Previously ZmpC has been linked to serotypes 11A, 8 and 33F of ST53 and ST62, both belonging to GPSC3 (Camilli et al., 2006; Gladstone et al., 2019; Hansen et al., 2021). In our study, the conservation within GPSC9 is presented. The protease importance for the success in dissemination and virulence of different GPSCs is something that can be of further interest to study due to its conservation in certain lineages recurring in pneumococcal disease like GPSC9 and most likely also GPSC3.

Irregularities between genotypic and phenotypic resistance were present although the concordance in general was high. One isolate carried *tetM* while still being sensitive, this has previously been noted and might be due to mutations within *tetM* not addressed in our study (Grohs et al., 2012). Overall β-lactam resistance showed a high correspondence pointing at the possibilities of using sequencing as a diagnostic tool for identifying resistance mechanisms in clinical isolates not detected by culture. Isolates included in the study were detected in routine clinical diagnostic testing. Isolated primarily from the nasopharynx (15/25) with an unknown indication for sampling. We thus cannot exclude that pneumococcus isolated might represent carriage strains rather than disease causing strains. However, they all represent highly resistant pneumococci that are present in the population of southern Sweden.

## Conclusion

The majority of the sequenced XDR and MDR pneumococcal isolates detected in Skåne County belonged to a limited number of GPSCs, primarily GPSC1, GPSC9 and GPSC10. Many *S. pneumoniae* were NVTs belonging to internationally widespread pneumococcal lineages of which many also cause invasive pneumococcal disease. The prevalence of XDR and MDR clones causing disease that are not targeted by current PCVs is of concern, such as those detected within GPSC10 and GPSC9. The most prevalent serotypes found are also common or increasing among IPD cases in Sweden post PCV10/13. Further surveillance is of importance as this may affect both treatment and prophylactic measures regarding IPD and mucosal pneumococcal disease.

## Data Availability Statement

The data presented in the article will be made available by the authors upon request. The genomic sequence data for the 25 isolates are deposited in the European nucleotide archive (https://www.ebi.ac.uk/ena) (ENA project accession: PRJEB41999).

## Ethics Statement

The study was approved by the local ethics committee (Regionala etikprövningsnämnden i Lund) 341 (approval no. 2012/286) and the ethical approval was updated (approval no. 2016/752) to include the current study period.

## Author Contributions

FU, JA, and KR contributed to conception of the study. LY, FU, H-CS, and KF developed methodology. LY and FU administered the project. LY, FU, and H-CS performed the bioinformatic analysis. LY wrote the first draft of the manuscript. All authors contributed to manuscript revision, read, and approved the submitted version.

## Funding

This study was financially supported by an unrestrained grant from Pfizer, the Anna and Edwin Berger Foundation (KR), Swedish Heart Lung Foundation (KR, #20180401), the Royal Physiographical Society (FU, Forssman’s Foundation), the Skåne County Council’s research and development foundation (KR), and Swedish Research Council (KR, #2019-01053).

## Conflict of Interest

JA and KR are participating in projects supported by Pfizer. KR is collaborating with Moderna and has been collaborating with GSK and been a scientific advisor to MSD and GSK. JA has received payments for lectures from AstraZeneca, GSK, MEDA and Pfizer. H-CS is involved with projects supported by Pfizer and has received payments for lecture from GSK.

The remaining authors declare that the research was conducted in the absence of any commercial or financial relationships that could be construed as a potential conflict of interest.

## Publisher’s Note

All claims expressed in this article are solely those of the authors and do not necessarily represent those of their affiliated organizations, or those of the publisher, the editors and the reviewers. Any product that may be evaluated in this article, or claim that may be made by its manufacturer, is not guaranteed or endorsed by the publisher.
